# The Perturbance of Microbiome and Gut-Brain Axis in Autism Spectrum Disorders

**DOI:** 10.3390/ijms19082251

**Published:** 2018-08-01

**Authors:** Greta Fowlie, Nicholas Cohen, Xue Ming

**Affiliations:** Department of Neurology, Rutgers New Jersey Medical School, 90 Bergen Street, DOC 8100, Newark, NJ 07103, USA; gfowlie22@gmail.com (G.F.); nicholas.cohen9@gmail.com (N.C.)

**Keywords:** microbiome, dysbiosis, gastrointestinal disorders, autism spectrum disorders, gut brain axis, leaky gut syndrome

## Abstract

Gastrointestinal problems have been documented in Autism Spectrum Disorder (ASD). Studies have found that these disturbances may be associated with an altered gut microbiome in ASD. Furthermore, in ASD, these alterations are implicated in increased gut permeability, or “leaky gut”, which allows bacterial metabolites to cross the gut barrier, impacting neurodevelopment during early childhood in susceptible subjects by way of gut-brain axis. In our review, we will discuss the interaction of gut microbiota and brain development in ASD and the signaling mechanisms underlying this interaction. We will also explore the potential for treatment of ASD by targeting the microbiome with probiotics. Finally, this paper will attempt to provide significance to the aggregation of the research in this area of research; providing our interpretations and assessments of future of this field.

Gastrointestinal (GI) problems have been documented in autism spectrum disorder (ASD). Almost half of children with ASD suffer from at least one GI symptom [1], and they tend to suffer more from GI symptoms as compared to their neurotypical counterparts [2], with diarrhea and constipation being the most common symptoms reported [3]. Additionally, recent studies show the severity of GI symptoms as being significantly correlated with the severity of autism symptoms [4,5,6]. These findings indicate a potential significant role of the intestinal environment contributing to the pathogenesis of ASD. This review will offer a concise overview of investigations in this area and attempt to give meaning to the aggregation of the research. 

The GI disturbances seen in ASD may be associated with an altered gut microbiome. The balance of microorganisms in the intestinal tract of ASD individuals has been found to differ from that of neurotypical individuals. In fact, the presence of autistic symptoms in children has been correlated with a less diverse gut microbiome, with one study finding significantly less carbohydrate degrading and fermenting bacteria of the genera *Prevotella*, *Coprococcus* and the unclassified *Veillonellaceae* in ASD microflora samples as compared to the neurotypical controls [7]. Another study reported that *Clostridium* spp. and enterococci were isolated more frequently from stool samples of autistic children as compared with controls, and there were quantitative differences observed mainly among staphylococci, *Candida* spp. and *Clostridium perfringens* [8]. Moreover, an increase in the Firmicutes/Bacteroidetes ratio was found in the gut microbiota of subjects with ASD [9]. The association of ASD and a number of microbial overgrowths, including various species of bacteria and *Candida*, have been further confirmed by independent studies over time [8,10,11,12]. In addition, Small Intestinal Bacterial Overgrowth has been correlated with ASD (see mini review [13]). Taken together, all these microbiome alterations may be associated with the increased gastrointestinal disturbances in individuals with ASD.

Beyond the gut microbiome, the very organic composition of the gut in ASD may be altered. Stool testing found lower levels of short chain fatty acids in children with ASD compared to the general population [4]. Another study found increased levels of IgA in stool samples of children with ASD compared to healthy children, suggesting the presence of gut immune abnormalities in ASD [14]. These alterations in gut composition may also be involved in the pathogenesis of ASD.

In addition to organic composition changes and microbiome imbalance, increased gut permeability or “leaky gut” is implicated in ASD [15,16,17]. Intestinal permeability, as measured by the lactulose/mannitol test, was found to be increased in patients with ASD [18]. Zonulin, an enzyme associated with regulation of intestinal permeability, was significantly increased in subjects with ASD and GI symptoms compared to healthy controls [19]. A similar study found that both the intestinal barrier and brain barrier may be impaired in ASD, with decreased levels of intestinal tight junction components and increased levels of claudin in the ASD brain compared to controls [20]. The “leaky gut” allows bacterial metabolites to readily cross the intestinal barrier, metabolites that do not naturally cross this barrier and are potentially neuroactive. Studies have shown evidence of increased metabolites in the urine and systemic circulation in ASD. There were increased gut bacterial metabolites in the urine of children with ASD and GI dysfunction [21]. Another study found that children with ASD had altered BPA metabolism, with increased BPA found in their urine [22]. Moreover, there is evidence of increased metabolites in the systemic circulation, as well, with increased serum endotoxin levels in subjects with ASD [23]. This “leaky gut” theory would offer a mechanism by which GI disturbances could play a role in neurodevelopment and cognition.

The presence of increased systemic metabolites in ASD is of importance due to the bi-directional relationship between the central nervous system and the gastrointestinal tract (the gut-brain axis) [24]. The “leaky gut”, through the neuroimmune, neuroendocrine, and autonomic nervous system, affects brain function, potentially contributing to the pathogenesis of ASD [17,25]. Therefore, it stands to reason that the altered metabolites detected in the urine and systemic circulation in ASD may play a part in affecting the brain and neurodevelopment.

With more research pointing to the importance of GI health in relation to neurological disorders, some studies have thus turned to targeting the microbiome for treatment of ASD. Nearly two decades ago, a study found that vancomycin temporarily improved behavior and communication in ASD [26]. More current research has focused on probiotics, which can normalize the altered gut bacterial ratio in ASD [5]. One case study demonstrated an improvement in core autism symptoms after long-term probiotic use [27]. Another study found that children with ASD who received a probiotic had significant improvement in behavioral symptoms, although there was no control arm in the study [28]. Parents of children with ASD who received a specific five strain probiotics Depro reported a significant improvement in bowel habits and behaviors measured by autism treatment evaluation checklist [28]. Kaluzna-Czaplinska showed an efficacy of probiotics in reducing *Candida* colonization in intestines in children with ASD [29]. More recently, fecal microbiota transplant in 18 children with ASD demonstrated an 80% improvement of GI symptoms and the effect lasted after discontinuation of an 8-week trial [30]. Antifungal treatment also demonstrated some efficacy in vivo [31]. While these studies are limited in their sample size and design, the use of probiotics for treatment of ASD with an attention to pathogenic biofilm [13] remains a promising avenue of investigation. 

While research into the gut-brain connection in autism still remains in its preliminary phases, there is a convincing body of evidence that suggests a relationship between gastrointestinal distress and autism. The severity of GI symptoms has been correlated with autism severity, strongly suggesting an interaction between the gut and the brain. GI distress in ASD may be due to an altered intestinal microbiome. The “leaky gut” and gut-brain axis indicates the mechanism by which these altered metabolites can enter the systemic circulation and directly affect neurodevelopment. However, further exploration into the treatment of microbiome imbalance in ASD is very much needed.

As the extent of research continues to grow, we hold that the importance of the interaction between the gut and the brain will become even more clear. Yet, even today, it is becoming evident that the gut, specifically the disturbance of it, plays an important role in certain neurological disorders including ASD.
